# Are predation rates comparable between natural and artificial open-cup tree nests in boreal forest landscapes?

**DOI:** 10.1371/journal.pone.0210151

**Published:** 2019-01-09

**Authors:** Katrine S. Hoset, Magne Husby

**Affiliations:** Section of Science, Nord University, Levanger, Norway; Fred Hutchinson Cancer Research Center, UNITED STATES

## Abstract

Nest predation studies often use artificial nests to secure sample sizes and nest distribution patterns that allow empirically testing differences in predation rates between ecological units of interest. These studies rely on the assumption that natural and artificial nests experience similar or consistent relative predation rates across ecological gradients. As this assumption may depend on several factors (for example differences in predator community, nest construction, parental care patterns), it is important to test whether artificial nests provide adequate and comparable estimates of predation rates to natural nests. In this study, we compare predation rates of above-ground natural open-cup nests, artificial nests and natural nests with artificial eggs along a forest gradient from edge to interior (interior, transition zone and edge) and within two nest visibility classes (visible and concealed). Our aim was to determine whether nest structure affects comparability between nest types along these ecological gradients in boreal forests. Our results indicated important contributions of nest type, nest visibility and location along the forest edge-interior gradient, but no variable had strong significant effects on predation rates, except exposure time that showed lower predation rates at longer exposure times. Predation rates in visible and concealed nests remained similar for all nest types, but not along the forest edge-interior gradient. Here, artificial nests showed much lower predation rates than natural nests, whereas natural nests with artificial eggs tended to have higher predation rates than natural nests. We conclude that artificial nests in boreal forests represent an adequate measure of relative nest predation risk in open-cup natural nests along some ecological gradients, but results on predation rates along forest edge-interior gradients obtained from artificial nests should be interpreted with care.

## Introduction

Nest predation is the most important factor causing reproductive failure (e.g., [1,2]), and therefore has been studied extensively. Nest predation studies often rely on the use of artificial nests to secure sample sizes and nest distribution patterns that allow empirically testing differences in predation rates between habitats, predator removal treatments or other units of interest [3–6]. Many of these studies rely on the assumption that natural and artificial nests experience similar predation rates, or that relative differences in predation rates between units are consistent between the two nest types [7]. However, the validity of this assumption has been questioned repeatedly (e.g., [8–15]). Although some studies comparing natural and artificial nests do suggest similarity in absolute predation rates [16–18], many studies show that artificial nests experience higher [4,15,19,20] or lower [9,10,12] predation rates than natural nests. These discrepancies in predation rates between natural and artificial nests have been attributed to several factors [3]. For example, different predator species may prey on artificial and natural nests [21], but also nest construction and visibility of artificial nests and characteristics of experimental eggs may increase or decrease the likelihood of predation [22,23]. Furthermore, absence of parental care in artificial nests can increase or decrease predator activity compared to natural nests [24], while increased nest density due to surplus of artificial nests may increase predation rates [25]. Finally, investigator activity may affect predation intensity of both artificial and natural nests [26,27]. Despite these biases, relative predation rates between natural and artificial nests can be comparable across ecological gradient, such as landscape fragmentation and forest patch size variation [4,28]. Regardless of the potential pitfalls of assuming artificial and natural nests provide similar information, artificial nests are still used in nest predation studies to ensure adequate sample sizes and nest distribution and to standardise conditions between nests. This highlights the need to identify situations where artificial nests provide adequate and comparable estimates of predation rates and where comparability between natural and artificial nests are misleading, as previously also suggested by Villard and Pärt [29].

In many of the studies where predation rates are compared between artificial and natural nest, the focus seem to be on the inconsistencies in treatment or experimental effect on predation rates rather than identifying the source of the difference. The latter is important for determining in which contexts artificial nests are a useful tool for estimating predation impact on bird species. For example, nest predation may be comparable between artificial and natural nests during the incubation period [12], or predation rate responses to habitat or forest edge differ between artificial and natural nests (e.g., [30]). The studies that have identified why predation rates differ between artificial and natural nests often highlight that predator identity differs between nest types [11,21,28]. However, in areas where corvids are the main predators, very similar nest predation rate between natural and artificial nests have been reported [16].

Different methods are employed to make artificial nest more similar to natural, e.g., using smaller clay or plasticine eggs instead of quail eggs [3,22,31–34], using old natural nests [8,35,36], old nest sites [37], or nesting densities and locations based on natural nest patterns [4,35,37,38]. However, little emphasis has been put into determining whether simulating natural nests in different manners actually leads to more comparable predation rates compared to natural nests.

Here we compare predation rates after 10 and 25 days exposure between natural nests and two types of artificial nests to determine whether nest structure (natural nest versus wire basket dressed with dry grass) affects comparability between nest types in boreal forests, while controlling for exposure time. Artificial nest types include artificial nests placed systematically in a study site at the same time as natural nests and old natural nests from the corresponding breeding season equipped with artificial eggs. Avian predators mainly cause nest predation in the study region with corvids dominating the avian predator community [39]. If differences between nest types are smaller when corvids are the most common predator (see [16]), we predict similar nest predation rates between nest types in our study. The comparability of predation rates between nest types was tested along ecological gradients known to affect nest predation rates in natural nests, namely nest visibility, and location along a forest edge-interior gradient (edge, transition zone and interior). We do not a priory expect any differences in nest predation rates in response to the ecological factors between nest types.

## Material and methods

### Study area

**Fig 1:**
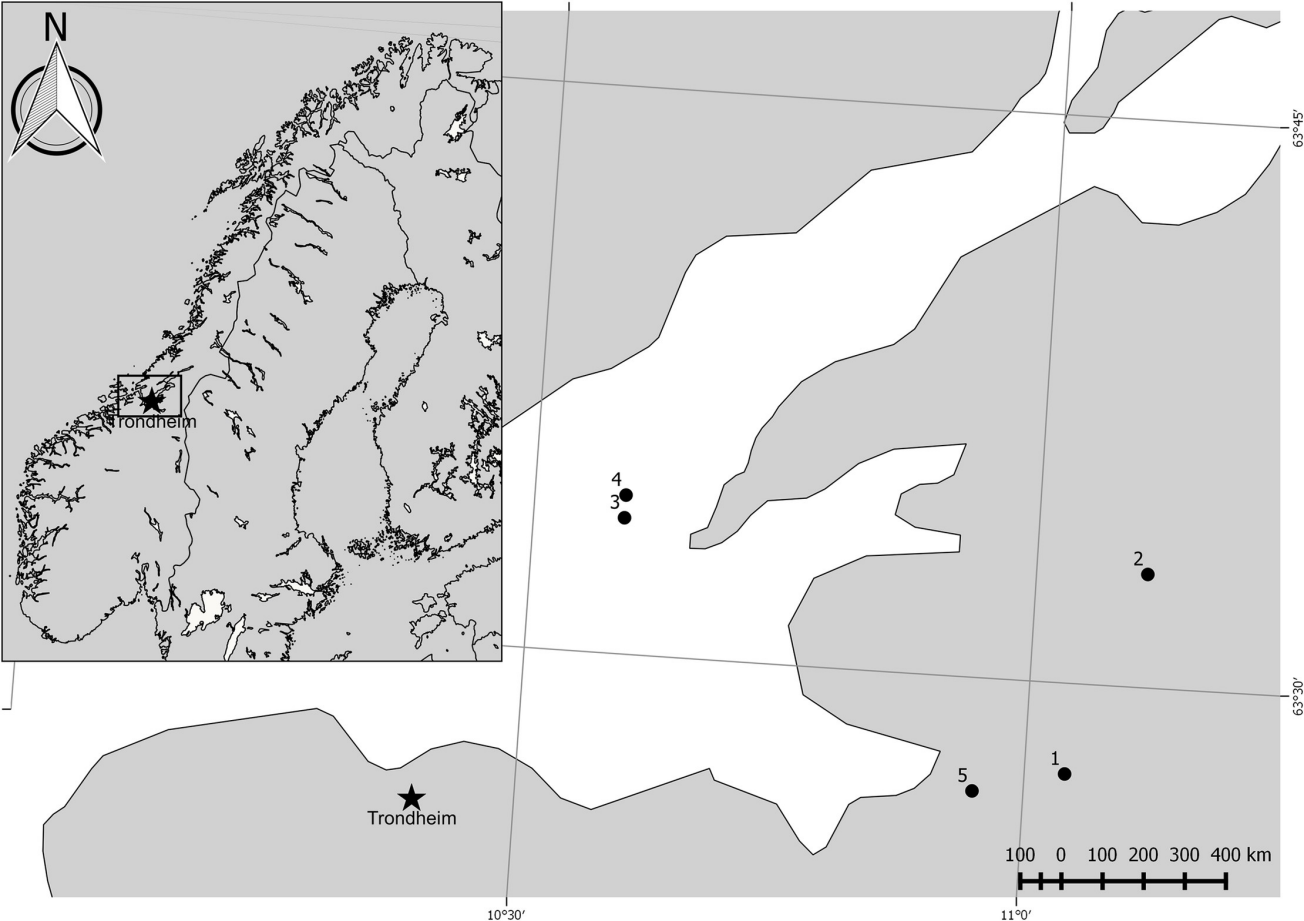
Map of study site locations relative to Northern Europe. Site numbers are provided in the map close up of the study area (see Table 1 for site details). For reference, the location of Trondheim city is represented by a star. Made with Natural Earth. Free vector and raster map data @ naturalearthdata.com.

All study areas are located in the western part of the mid-boreal forest zone in Norway. It is a mosaic landscape with forest, forest patches, farmland, lakes and ocean, mires and human habitations. Potential predators at the sites include different corvid species, mustelids, squirrels, foxes and badgers (see details in [39]). The study includes five sites located in forest patches, which were monitored between 2004 and 2006 (percentage forest cover and arable land within a 5.5 km^2^ area at each site is presented in Table 1. Land cover percentages were obtained from OSM Landuse Landcover based on OpenStreetMap-data (osmlanduse.org [40])). Previous studies suggest that predation rates on both natural and artificial nests in this region are mostly similar between different study years [39], using data from multiple years should therefore not introduce too much bias due to between year variation in predation rates. Sites were distributed between 63.4 and 63.6^o^N, and 10.5 and 11.2^o^E within central Norway (Table 1, Fig 1). The forests are dominated by Norwegian spruce *Picea abies* (59% of total forest in Trøndelag county between 2004 and 2008, data from Statistics Norway: www.ssb.no/en), with inclusion of Scots pine *Pinus silvestris* and deciduous tree species (22% pine and 19% deciduous trees between 2004 and 2008, data from Statistics Norway: www.ssb.no/en). We defined forest locations along a gradient from interior to edge: forest interior (> 50m from the forest edge), edge (within 2m from the forest edge) and transition zone between forest interior and edge (2 – 50m from edge inside the forest). The definition of forest interior at distance > 50 m was based on the meta-analysis results of Batáry and Báldi [30], which showed that edge effects were negligible beyond 50 m. The choice of including a transition zone was based on the same study, which showed that edge effects may be strong at least up to 25 m from the edge. Forest edges were mainly natural edges along agricultural land, with maximum 3 nests across sites located along clear-cuts.

**Table 1:** Distribution and number of nest types between sites.

site no	% forest / arable	mainland/island	natural	artificial	nat w/artegg	total
1	16.3 / 75.6	mainland	1	30	27	**58**
2	72.7 / 1.6	mainland	20	8	12	**40**
3	51.4 / 41.4	island	12	14	11	**37**
4	44.3 / 49.7	island	49	96	40	**185**
5	13.5 / 38.5	mainland		30	17	**47**

Nest types include natural nests, systematically placed artificial nests (artificial), and natural nests with artificial eggs (nat w/artegg). Total nest numbers, position of sites on mainland or island and % cover of forest and arable land are also provided. Land use data are percentages of available land area, and excludes sea as water bodies, but includes lakes and rivers. Percentages are provided for a land area of ca 5.5 km^2^ per site, which defines an area that provides representative information on surrounding land cover types while avoiding extensive overlap between sites. Percentage land cover was obtained from OSM Landuse Landcover based on OpenStreetMap-data (osmlanduse.org [40]) and excludes land areas that are not provided with landuse tags in OpenStreetMap. The reported values therefore represent approximate estimates and not absolute values.

### Experimental design

We monitored predation occurrence on natural and artificial eggs (see details below) after 10 and 25 days from three types of nests; natural nests, systematically placed artificial nests (hereafter artificial nests) and natural nests with artificial eggs. Exposure of 10 days represented the exposure time used in most artificial nest studies by the research team (see [39,41]), while exposure of 25 days represent the average incubation plus nestling period of natural nests. All nests used in this study were classified into three categories of visibility based on how visible nests were at different distances: 1) high cover, nests hardly visible at 0.5m distance, 2) medium cover, nests visible at 1m, and 3) low cover, nests visible at 1-5m. As there were very few natural nests (n = 5) and natural nests with artificial eggs (n = 4) that were classified as high cover, we combined the two classes “high” and “medium” cover to one class and operate now with concealed and visible nests. Only natural nests and natural nests with artificial eggs originating from birds of the families Turdidae and Fringillidae were used in this experiment, as they are the most common species in the habitat used for this study.

**Natural nests** were found before and during egg laying (nests active during May and June) and the fate of individual nests were monitored until fledging (see Table 2 for a complete species list). Natural nests located within fieldfare *Turdus pilaris* colonies were not included while fieldfares were breeding. This was done because i) their colonial breeding and intensive nest defence both against nest predators and human intruders can disturb experiments (e.g., [42,43]), ii) in most colonies we had data only on fieldfare nests, and iii) we did not have natural nests with artificial eggs within colonies or colonies within island sites. Most nests were located from 0.5 m to 6 m above ground (97%), with a few nests located below 0.5 meter or above 6 meter (3%).

**Table 2:** Species list.

Species	Scientific name	Family	Natural nests	Natural nests with artificial eggs
Eurasian blackbird	*Turdus merula*	Turdidae	1	1
Fieldfare	*Turdus pilaris*	Turdidae	0	8
Redwing	*Turdus iliacus*	Turdidae	26	34
Song thrush	*Turdus philomelos*	Turdidae	8	6
Common chaffinch	*Fringilla coelebs*	Fringillidae	1	17
European greenfinch	*Chloris chloris*	Fringillidae	37	29
Twite	*Linaria flavirostris*	Fringillidae	3	3
Eurasian bullfinch	*Pyrrhula pyrrhula*	Fringillidae	1	0

List of bird species (common and scientific names) with taxonomic family and the number of natural nests and natural nests with artificial eggs represented by each species.

**Artificial nests** were distributed in May, June or July with a minimum distance of 100 m between each nest. All nests were open-cup nests with 11 cm outer diameter placed on the in trees 1–1.5m above the ground within all forest location categories, since all natural nests and natural nests with artificial eggs were located in trees. Nests were designed for a common bird species with similar sized nests as the artificial nests and do not model a specific bird species. This allows us to compare predation rates of most natural bird nests available at each site. Any potential effects of bird species on nest design are checked by adding natural nests with artificial eggs as a separate nest type (see description below). Nests were made of wire baskets lined with dry grass and/or moss, and were attached to tree branches with iron wire. The metal was visible from beneath in tree nests, but completely hidden by nesting material from above, and therefore the appearance was quite similar to a natural nest.

**Natural nest with artificial eggs** were natural nests predated during or after egg laying or with successful fledging, in which we placed artificial eggs after the main breeding season (July, all natural nests used were active during May and June). Natural nests abandoned before egg-laying were not included since natural nests with artificial eggs might be abandoned for other reasons than nest predation.

In artificial nest and natural nests with artificial eggs, we put one fresh quail *Coturnix coturnix* egg and one plasticine egg. The plasticine eggs were a mixture of grey and green plasticine and had the similar size and shape as the quail eggs. We attached the plasticine egg with a metal wire with a barb inside the egg, and the metal wire was attached to a branch under the nest or in the surrounding vegetation. The plasticine and quail eggs were similar in size to many relatively common species in the areas surrounding the study sites, nesting in trees (e.g., thrushes and common wood pigeon *Columba palumbus*), and the most numerous nest predators in our areas will easily predate the actual egg sizes. A nest was considered predated if at least one of the eggs had been removed or damaged by a predator.

A few plasticine eggs had marks made by small mammals (different mouse or vole species), snails or tits, and a few quail eggs had small scratch marks without being destroyed. We did not consider these groups as predators because they cannot destroy quail eggs and are not potential predators for eggs at this size [22,32,33,44]. Therefore, the few eggs with marks only from small mammals, snails or tits were recorded as not being predated. Thus, we also avoided the potential issue the great multi-annual variation in abundance of small mammals in boreal forests can have on predation rates (e.g., [45,46]), especially in nests not guarded by parents. However, small mammals may predate small eggs from for example warblers [47], but the importance of small mammals as nest predators are probably overestimated in artificial nest experiments when plasticine is used [48]. Disregarding these nests as predated would potentially lead to smaller differences in predation rates between natural nests and artificial nests in our analyses, which makes our inferences conservative at worst. We have no grounds to believe that this would affect the response to ecological factors included in the analysis, and should therefore not affect the relative comparability between artificial and natural nests.

We did not use rubber boots or gloves to avoid leaving scents at the nest, since several studies show that there are small chances for predators to learn, by smell or visually, to associate human scent to artificial nests [44,49]. This choice should not lead to large biases, since the same method was used in all sites and all years. Nest locations were not marked. No specific permissions were needed for the fieldwork since we only observe bird’s nests and use artificial nests to assess predation rates. The field work did not involve endangered or protected species.

### Data analyses

Predation rates in natural and artificial nest types were compared using a logistic regression fitted as a generalised linear mixed model (GLMM) within a Bayesian framework (package **rstandarm** [50]) in R statistical software (version 3.4.3, [51]). We use a logistic regression model for this analysis since the posterior resampled distribution of a binomial regression model with a complementary log-log link and exposure time as offset showed poor fit (see details on the model building procedure in S1 Appendix). Predation events (0 for survived and 1 for predated) recorded after 10 or 25 days exposure was entered as the dependent variable in the logistic regression. Some nests were checked after both 10 and 25 days exposure. Of these, nests predated before 10 days were defined to have 10 days exposure, whereas remaining nests were defined to have 25 days exposure. Nest type (natural, artificial and natural with artificial eggs), forest location (forest interior, edge and transition zone), visibility (extra cover, good cover and visible), and the interactions between nest type and the other main effects were added as predictor variables. To control for the potential confounding effects of site location on mainland or island, nest activity month and taxonomic family (thrushes and finches), island, month, family and the interaction between family and nest type were included as predictor variables. Finally, to control for differences in exposure time between nests, we added z-transformed exposure as a predictor variable. The variable exposure time was z-transformed to improve model convergence and obtain reliable parameter estimates (models with non-transformed exposure time provided predation estimates close to 1). Site no was used as a random effect in the model.

As weakly informative prior distributions for the intercept and the vector of regression coefficients we chose the default normal distribution with mean = 0 and standard deviation = 1. We also chose to use the default prior for the covariance structure, which is developed to be robust for common applied regression problems [52]. The covariance matrix is decomposed into a vector of variances and a correlation matrix, which summarises the covariation of slopes and intercepts in a standardised form. Default settings describe a joint uniform prior for the correlation matrix. The vector of variances is set equal to the product of a simplex vector described by a symmetric Dirichlet prior. With the default settings of a concentration parameter at 1, this prior is jointly uniform over the space of simplex vectors.

Posterior distributions were obtained by fitting the model described above with function **stan_glmer** in package **rstanarm**, which uses a Hamiltonian Markov Chain Monte Carlo algorithm to obtain samples drawn from the posterior distribution. Default settings fits four Markov chains with 2000 iterations each (thinning = 1), half of which are discarded as a warm-up used to give the algorithm time to find the target posterior area. We assessed model fit by performing posterior predictive checks and sensitivity analysis following suggestions by Gelman et al. [53]. Model convergence, effective posterior sample size, Monte Carlo standard error and posterior predictive checks were performed using the **shinystan** R package [54], which provides a graphical user interface for exploring models fit using MCMC-techniques. All predictors had Ȓ = 1, Monte Carlo standard error < 0.1 and number of effective samples > 1210. In the sensitivity analysis, we tested the impact of choice for prior distributions on intercept and the vector of regression coefficients (see S1 Appendix for further details on the sensitivity analysis and model evaluation).

The means of simulated values from the joint posterior distribution of the model parameters were used as estimates, and the 2.5% and 97.5% quantiles as the lower and upper limits of the 95% credible intervals. Posterior probabilities of linear predictor effects being larger (or smaller) than zero were calculated by the proportion of simulated values from the posterior distribution being higher (or smaller) than zero. To simplify language in the results, we declare an effect to be significant if the posterior probability of being positive (or negative) is larger than 0.97, and we declare an effect to signify a trend if the posterior probability of being positive (or negative) is larger than 0.75. These values correspond to the limits of the 95% and 50% credible intervals, respectively, for the model not containing zero.

## Results

**Fig 2:**
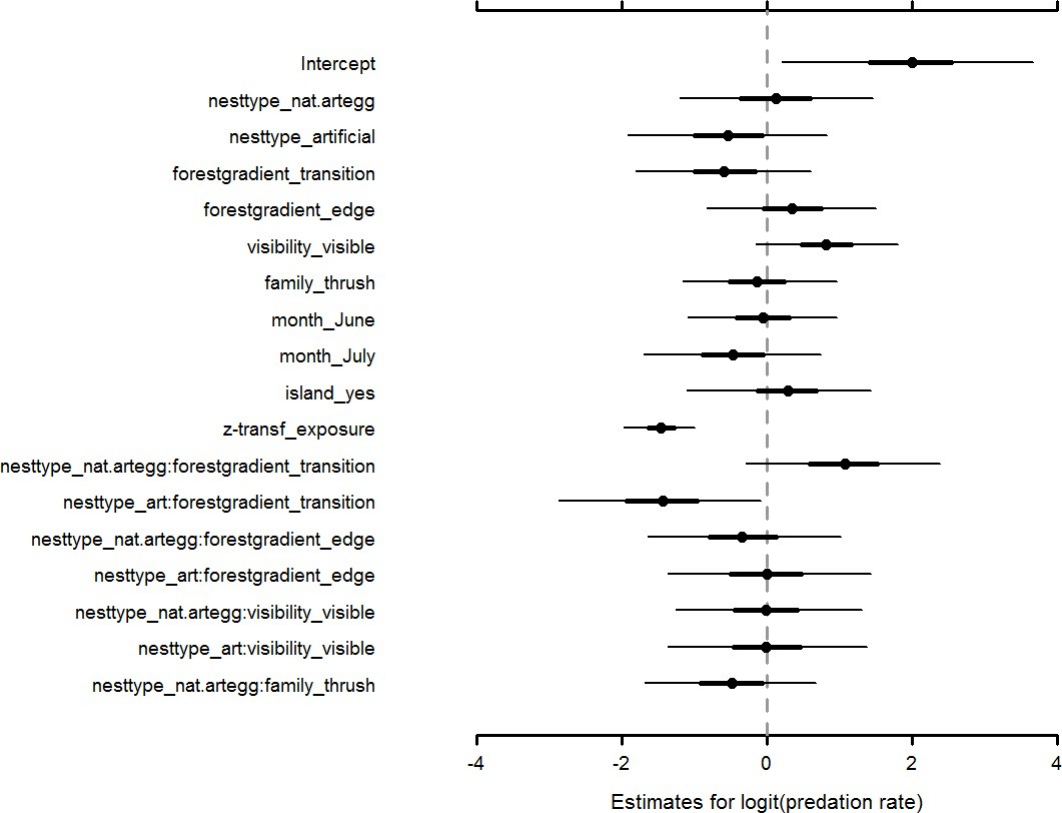
Parameter effect estimates in logit scale. Effect estimates of each main and interaction effect (fixed factor) in the model. For each effect estimates, the thick black line represent the 50% credible interval and the thin black line represent the 95% credible interval. A grey stippled vertical line highlights the location of the zero line to ease readability. Effects for which the thick 50% credible interval crosses the zero-line are interpreted to not be significant, effects for which the thin 95% credible interval line crosses zero are interpreted as representing trends, while effect for which the credible line does not cross zero are interpreted as being significant. Intercept represents well covered natural nests located in the forest interior and observed in May. Abbreviations: art = artificial nests, nat.artegg = natural nests with artificial egg, z-tranf = z-transformed.

Observed average predation rates were high (Table 3). The intercept value from the logistic regression model (Table 4) corresponded reasonably well with the observed predation rates with a back-transformed estimate of predation rate at 88.1%. Of the main predictor effects, only exposure time showed a significant negative effect with nests exposed for 25 days having lower predation rates than nests with a 10-day exposure time (Fig 2, Table 4). However, there was indications that nest predation rates depended on nest type, location along the forest gradient, nest visibility and observation month (Fig 2). Artificial nests had a probability of 0.77 of having lower nest predation rates than natural nests, and the probability of predation rates being lower in the transition zone than in the forest interior was 0.83 (Table 4). Visible nests was 95% more likely to have higher predation rates than covered nests, and nest predation was 77% more likely to be higher in July compared with May (Table 4).

**Table 3:** Observed predation rates for different nest types according to location along the forest edge-interior gradient and nest visibility.

Nest type	Forest gradient	Predation rates (mean ± se) in covered nests	Predation rates (mean ± se) in visible nests
Natural	Interior	0 ± 0	1 ± 0
	Transition	0.833 ± 0.167	0.667 ± 0.211
	Edge	0.864 ± 0.052	0.857 ± 0.097
Natural w/art. egg	Interior	0 ± 0	0.824 ± 0.095
	Transition	0.75 ± 0.25	0.844 ± 0.065
	Edge	0.857 ± 0.060	0.571 ± 0.202
Artificial	Interior	0.938 ± 0.063	1 ± NA
	Transition	0.563 ± 0.128	0.733 ± 0.118
	Edge	0.813 ± 0.101	1 ± 0

**Table 4:** Posterior effect estimates and credible intervals for fixed and random effects.

Parameters	Mean	2.5%	97.5%	P(β > 0)
**intercept (natural nest, forest interior, covered)**	**1.974**	**0.208**	**3.671**	**0.984**
nesttype_nat.artegg	0.127	-1.206	1.466	0.579
***nesttype_artificial***	***-0*.*533***	***-1*.*915***	***0*.*834***	***0*.*227***
***forest*.*gradient_transition***	***-0*.*589***	***-1*.*808***	***0*.*602***	***0*.*168***
forest.gradient_edge	0.351	-0.822	1.510	0.726
***visibility_visible***	***0*.*822***	***-0*.*157***	***1*.*805***	***0*.*947***
family_thrush	-0.130	-1.159	0.962	0.400
month_June	-0.060	-1.090	0.962	0.461
***month_July***	***-0*.*469***	***-1*.*701***	***0*.*742***	***0*.*226***
island_yes	0.255	-1.102	1.437	0.686
**z-transformed exposure**	**-1.463**	**-1.971**	**-0.995**	**0**
***forest*.*gradient_transition*:*nesttype_nat*.*artegg***	***1*.*065***	***-0*.*283***	***2*.*391***	***0*.*940***
**forest.gradient_transition:nesttype_artificial**	**-1.448**	**-2.864**	**-0.087**	**0.018**
forest.gradient_edge:nesttype_nat.artegg	-0.327	-1.644	1.018	0.313
forest.gradient_edge:nesttype_artificial	-0.001	-1.359	1.435	0.501
*Visibility_visible*:*nesttype_nat*.*artegg*	-0.004	-1.259	1.318	0.492
Visibility_visible:nesttype_artificial	0.004	-1.369	1.384	0.495
***Family_thrush*:*nesttype_nat*.*artegg***	***-0*.*489***	***-1*.*679***	***0*.*679***	***0*.*219***
Random effect site 1	0.104	-0.814	1.463	
Random effect site 2	0.423	-0.369	1.893	
Random effect site 3	-0.272	-1.443	0.700	
Random effect site 4	0.404	-0.479	2.083	
Random effect site 5	0.184	-0.741	1.550	
Sigma	0.603	0	3.388	
Mean_PPD	0.806	0.743	0.862	
Log-posterior	-127.634	-136.642	-121.062	

Mean effect estimates with 95% credibility interval of nest type, forest gradient location, nest visibility, taxonomic family, month, island and exposure time on nest survival rates. For fixed effects, we show the probability that effect slopes are above zero. Slopes of effect estimates with a probability between 2.5% and 97.5% of being above zero are highlighted with bold text; estimates with probability between 25% and 75% of being above zero are highlighted with bold italic text. The intercept represents baseline categories of predictor levels, which are listed in parentheses in order nest type, forest gradient location and visibility, for which other predictor levels (factorname_levelname) are compared with.

Relative differences in nest predation rates seemed to be similar between nest types between levels of nest visibility (Fig 2, Table 4). However, nest predation rates were not fully comparable within the forest gradient. Although nest predation rates seem to be similar between nest types when comparing predation rates in the forest interior and at the forest edge, both artificial nests and natural nests with artificial eggs showed different predation rates in the transition zone (Fig 2). Natural nests with artificial eggs tended to have higher predation rates than natural nests in the transition zone (P(β > 0) = 0.94), whereas predation rates in artificial nests were significantly lower compared with natural nests in the transition zone (Table 4). The only other interaction with evidence of significance was the interaction between nest type and taxonomic family. Natural nests with artificial eggs belonging to thrushes had a probability of 0.78 of being lower than natural nests (with artificial eggs) of finches (Table 4).

## Discussion

Predation is a major cause of nest failure in birds [1,2] and can have an enormous impact on life history evolution [55]. An interplay of several factors causes spatial and temporal variation in nest predation rates, involving characteristics of the birds themselves, the predators, and the surrounding habitat. Behaviour of prey bird species can affect predation rates through choice of nesting sites [37], nest density [49,56], nest concealment [57–59] and parental activity at the nest [60,61]. Predator characteristics important for nest predation risk include for example predator abundance and breeding phenology [49,62], community composition [48,63] and availability of alternative prey [64,65]. Yet again, factors related with landscape features, such as habitat differences, fragmentation or edge effects, can have strong impact on local predator abundances and predation pressure [30,66,67]. Often, patterns of variation in nest predation are evaluated based on studies using artificial nests instead of natural nests. In all the above-mentioned situations that determine nest predation rates, artificial nests may or may not experience the same patterns of predation as natural nests [12,15,17,18]. In this paper, we have evaluated how well artificial nests represent nest predation rates in natural nests according to nest visibility and location along a forest edge-interior gradient.

The findings of this study reveal that estimated nest predation rates were high, corresponding to high observed predation rates, but similar to predation rates observed for artificial tree nests in the study region in some years [39]. Nest predation rates tended to differ according to nest type (natural, natural with artificial eggs, and artificial), location along a forest gradient (interior, transition, edge), and nest visibility. Of the controlling factors, nest predation rates tended to differ according to month of observation (lower predation in July than May) and differed depending on exposure time (lower predation rates in nests exposed for 25 days than nests exposed for 10 days), whereas predation rates in natural nests with artificial eggs tended to be lower in nests of thrushes compared with nests of finches. When controlling for exposure time, predation rates of artificial nests tended to be lower than predation rates in natural nests, which correspond to results from other studies [9,10,12]. Observed predation rates (exposure time not controlled for) were similar between natural and artificial nests, highlighting the importance of controlling for exposure time when assessing nest predation rates. Interestingly, natural nests with artificial eggs showed similar nest predation rates as natural nests, even though artificial eggs were added to natural nests in July, after the breeding season of the original residents. Relative timing of the treatment can affect the comparability of predation rates between natural and artificial nests due to seasonal variation in predation rates (e.g., [68,69]), and predation rates of artificial nests in our study system follow a seasonal pattern with peaks in June/July [41]. We could therefore expect lower predation rates in natural nests with artificial eggs compared with natural nests based on the timing of exposure. One possible explanation for our result is that some of the natural nests used in the experiment were predated before reuse, which could bias our results towards higher predation rates if predators are more likely to visit these nests later in the season. Although nest design can be an important feature determining predation rates (reviewed in [70]), the likelihood of predation in re-used nests that already experienced predation does not seem to have been investigated. Another option is that the expected pattern of lower predation rates due to season only apply to thrushes and not to finches, given the trend in the interaction effect between nest type and taxonomic family (technically between natural nests and natural nests with artificial eggs as artificial nests were not labelled according to taxonomic family). All nest types varied similarly with respect to nest visibility, but not according location along the forest edge-interior gradient.

We found predation rates to be significantly lower after 25 days exposure than after 10 days exposure, which seems counterintuitive. However, this result is partly caused by study design and data management choices related with categorisation of nests within 10- or 25-days exposure. Some nests were observed after both 10 and 25 days, of which nests predated before 10 days were categorised as being exposed for 10 days and the remaining nests were treated as being exposed for 25 days. This causes a skew in predation rate estimation towards being higher during the 10-day period as predated nests observed both exposure periods are censored out and not included in the estimations of predation rate after 25-days exposure. This is evident if we redefine all those nests as being exposed for 25 days, at which point the effect of exposure is largely reduced (S1 Fig). Therefore, our result may suggest that predation rates are higher during a time period representing the incubation period compared with predation rates during the nestling period, assuming if nests that survive during the first 10 days have lower risk of being predated later. However, most of the nests within the 10-day exposure category are artificial nests (or among nests predated before 10 days, S1 Table), whereas natural nests and natural nests with artificial eggs are more evenly distributed between the exposure time categories (S1 Table). Under this scenario, the results may indicate that artificial nests experience higher predation rates than natural nests when differences in exposure time are not controlled for. Note, however, that when redefining all double-observed nests as having exposure time of 25 days, predation rates of artificial nests do not differ significantly from rates in natural nests but the mean point estimate is positive rather than negative (S1 Fig). Alternatively, the remaining small effect of exposure time may relate with, for example, parental activity at natural nests that reduces the relative predation risk in these nests compared with artificial nests. Parental and nestling activity at nests can both increase and decrease predation rates in artificial nests compared with natural nests [24,71], and some bird species adjust their activity at the nest dependent on nearby predator activity [72].

Nest predation rates tended to be lower in visible nests compared with concealed nests. This is to be expected since nest visibility is an important determinant for predation risk in many systems [73–76], and is linked with higher predation rates of visually searching predators [73], which are previously documented to dominate predation events in our study region [39,41]. This tendency for predation rates to depend on nest visibility probably reflect how easily predators can find the nests. However, the difference in predation rates between visibility classes remain similar between nest types, and the posterior estimated mean interaction effect was close to zero for both natural nests with artificial eggs and artificial nests. Also other studies have found relative nest predation rates between natural and artificial nests to respond similarly to nest concealment as we found in our study [20]. Relative predation rates between nest types thus seem to be comparable for different levels of this important ecological factor.

Along the forest edge-interior gradient, nest predation rates tended to be lower in the forest transition zone than the forest interior, but were similar between the forest interior and the edge. These results suggest weak or no evidence for edge effects in our study area when comparing the two extreme ends of the gradient. This result correspond with results from other studies in Europe [32,76,77], although some studies, in Europe and elsewhere, have found edge effects [66,73]. The discrepancies in results probably depend on regional or site-specific differences in main predator class and diversity [32,78,79], habitat type and type of edges investigated [30]. However, although predation rates for all nest types followed similar patterns between forest interior and forest edge, predation rates in artificial nests were markedly lower in the transition zone compared with natural nests and natural nests with artificial eggs tended to have higher predation rates than natural nests in the transition zone. Our transition zone (within 2–50 m distance from forest edge) correspond to an area where edge effects should be expected [30,73], suggesting that studies with artificial nests using distance within 50 meter from edge may show misleading patterns of predation rates compared with natural nests. Different conclusions regarding edge effects between studies could therefore, to some extent, be explained by the mixed use of natural and artificial nests in these studies, and different definitions of the edge zone.

## Conclusion

This study indicates that both actual and relative predation rates were comparable along some of the ecological gradients explored, which correspond to the findings of other studies [4,16–18]. A clear exception to this concerned predation rates along the forest edge-interior gradient. Although natural nests with artificial eggs displayed similar absolute predation rates as natural nests, whereas artificial nests tended to have lower predation rates, both artificial nest types showed deviating predation rates from natural nests in the forest transition zone. We therefore suggest that studies using artificial nests to explore differences in predation rates along forest edge-interior gradients should be interpreted with care, especially at distances of ca 2–50 meter from edges. Comparability of predation rates between natural and artificial nests may still be valid when comparing the true forest edge (within 2 meters from the edge line) with the interior of a patch, although the comparability does not hold along the whole gradient for any artificial nest type tested here. Consistency and comparability of predation rates between natural and artificial open-cup nests often depend on an interaction between nest type and the ecological condition being tested [80], as we also show in this study. We therefore stress the importance of testing the comparability between artificial and natural nests when planning to use artificial nests to assess predation rates in new areas or in ecological contexts where this relationship has not been tested before.

## Supporting information

S1 Appendix Model building and checking. Model building procedure with model checking and evaluation from original binomial regression model with complementary log-log link and exposure time as offset. Including r code used for analysis.
(PDF)

S1 File Data file with all observations used for analysis. (CSV)

S1 Fig Dependency of exposure time effect on categorization of exposure time in nests observed at both 10 and 25 days. Effect sizes of predictor variables explaining variation in nest predation rates based on dataset where all nests observed at both 10 and 25 days were categorized as having 25 days exposure time.
(DOCX)

S1 Table Distribution of nest types according to exposure time. The table highlights how nest belonging to different nest type categories are distributed between 10 and 25 days exposure time in the dataset used for analysis in the paper, and for two alternative datasets where all nests observed after both 10 and 25 days are defined as having exposure time of either 10 days (Adjusted exposure time 10 days) or 25 days (Adjusted exposure time 25 days). See S1 Fig how the difference in categorization affects predictor estimates, and especially the effect of z-transformed exposure time.
(DOCX)

## References

[1] Ricklefs RE. An analysis of nestling mortality in birds. Smithson Contr Zool. 1969;9: 1–48.

[2] Seibold S, Hempel A, Piehl S, Bässler C, Brandl R, Rösner S, et al Forest vegetation structure has more influence on predation risk of artificial ground nests than human activities. Basic Appl Ecol. 2013;14: 687–693. 10.1016/j.baae.2013.09.003

[3] Major RE, Kendal CE. The contribution of artificial nest experiments to understanding avian reproductive success: a review of methods and conclusions. Ibis. 1996;138: 298–307. 10.1111/j.1474-919X.1996.tb04342.x

[4] Wilson GR, Brittingham MC, Goodrich LJ. How well do artificial nests estimate success of real nests. Condor. 1998;100: 357–364.

[5] Weidinger K. How well do predation rates on artificial nests estimate predation on natural passerine nests? Ibis. 2001;143: 632–641. 10.1111/j.1474-919X.2001.tb04891.x

[6] Fontaine JJ, Martel M, Markland HA, Niklison AA, Decker KL, Martin TE. Testing ecological and behavioral correlates of nest predation. Oikos. 2007;116: 1887–1894. 10.1111/j.2007.0030–1299.16043.x

[7] Wilcove DS. Nest Predation in Forest Tracts and the Decline of Migratory Songbirds. Ecology. 1985;66: 1211–1214. 10.2307/1939174

[8] Martin TE. Artificial Nest Experiments: Effects of Nest Appearance and Type of Predator. The Condor. 1987;89: 925–928. 10.2307/1368547

[9] Willebrand T, Marcström V. On the danger of using dummy nests to study predation. The Auk. 1988;105: 378–379. 10.2307/4087508

[10] Roper JJ. Nest Predation Experiments with Quail Eggs: Too Much to Swallow? Oikos. 1992;65: 528–530. 10.2307/3545570

[11] Davison WB, Bollinger E. Predation Rates on Real and Artificial Nests of Grassland Birds. The Auk. 2000;117: 147–153.

[12] Vander Haegen WM, Schroeder MA, DeGraaf RM. Predation on Real and Artificial Nests in Shrubsteppe Landscapes Fragmented by Agriculture. The Condor. 2002;104: 496–506.

[13] Zanette L. What do artificial nests tells us about nest predation? Biol Conserv. 2002;103: 323–329. 10.1016/s0006-3207(01)00143-4

[14] Chalfoun AD, Thompson FR, Ratnaswamy MJ. Nest Predators and Fragmentation: a Review and Meta-Analysis. Conserv Biol. 2002;16: 306–318. 10.1046/j.1523-1739.2002.00308.x

[15] Burke DM, Elliott K, Moore L, Dunford W, Nol E, Phillips J, et al Patterns of nest predation on artificial and natural nests in forests. Conserv Biol. 2004;18: 381–388. 10.1111/j.1523-1739.2004.00014.x

[16] Yahner RH, DeLong CA. Avian Predation and Parasitism on Artificial Nests and Eggs in Two Fragmented Landscapes. Wilson Bull. 1992;104: 162–168.

[17] Major RE, Pyke GH, Christy MT, Gowing G, Hill RS. Can Nest Predation Explain the Timing of the Breeding Season and the Pattern of Nest Dispersion of New Holland Honeyeaters? Oikos. 1994;69: 364–372. 10.2307/3545849

[18] Kurucz K, Bertalan L, Purger JJ. Survival of blackbird (*Turdus merula*) clutches in an urban environment: experiment with real and artificial nests. North-West J Zool. 2012;8: 362–364.

[19] Storaas T. A Comparison of Losses in Artificial and Naturally Occurring Capercaillie Nests. J Wildl Manag. 1988;52: 123–126. 10.2307/3801071

[20] King DI, DeGraaf RM, Griffin CR, Maier TJ. Do Predation Rates on Artificial Nests Accurately Reflect Predation Rates on Natural Bird Nests? J Field Ornithol. 1999;70: 257–262.

[21] Thompson FR, Burhans DE. Differences in Predators of Artificial and Real Songbird Nests: Evidence of Bias in Artificial Nest Studies. Conserv Biol. 2004;18: 373–380. 10.1111/j.1523-1739.2004.00167.x

[22] Haskell DG. Forest fragmentation and nest predation: are experiments with Japanese quail eggs misleading? The Auk. 1995;112: 767–770.

[23] Rangen SA, Clark RG, Hobson KA. Visual and olfactory attributes of artificial nests. The Auk. 2000;117: 136–146. 10.1642/0004-8038(2000)117[0136:VAOAOA]2.0.CO;2

[24] Cresswell W. Nest Predation Rates and Nest Detectability in Different Stages of Breeding in Blackbirds *Turdus merula*. J Avian Biol. 1997;28: 296–302. 10.2307/3676942

[25] Martin TE. On the advantage of being different: nest predation and the coexistence of bird species. Proc Natl Acad Sci. 1988;85: 2196–2199. 16593919 10.1073/pnas.85.7.2196 PMC279956

[26] Major RE. The effect of human observers on the intensity of nest predation. Ibis. 1990;132: 608–612. 10.1111/j.1474-919X.1990.tb00285.x

[27] Rotella JJ, Taper ML, Hansen AJ. Correcting nesting-success estimates for observer effects: maximum-likelihood estimates of daily survival rates with reduced bias. The Auk. 2000;117: 92–109. 10.1642/0004-8038(2000)117[0092:CNSEFO]2.0.CO;2

[28] Pärt T, Wretenberg J. Do artificial nests reveal relative nest predation risk for real nests? J Avian Biol. 2002;33: 39–46. 10.1034/j.1600-048X.2002.330107.x

[29] Villard M-A, Pärt T. Don’t Put All Your Eggs in Real Nests: a Sequel to Faaborg. Conserv Biol. 2004;18: 371–372. 10.1111/j.1523-1739.2004.00485.x

[30] Batáry P, Báldi A. Evidence of an edge effect on avian nest success. Conserv Biol. 2004;18: 389–400. 10.1111/j.1523-1739.2004.00184.x

[31] Møller AP. Nest Site Selection across Field-Woodland Ecotones: The Effect of Nest Predation. Oikos. 1989;56: 240–246. 10.2307/3565342

[32] Nour N, Matthysen E, Dhondt AA. Artificial nest predation and habitat fragmentation: different trends in bird and mammal predators. Ecography. 1993;16: 111–116.

[33] Bayne EM, Hobson KA, Fargey P. Predation on artificial nests in relation to forest type: contrasting the use of quail and plasticine eggs. Ecography. 1997;20: 233–239. 10.1111/j.1600-0587.1997.tb00366.x

[34] Willson MF, Morrison JL, Sieving KE, De Santo TL, Santisteban L, Díaz I. Patterns of Predation Risk and Survival of Bird Nests in a Chilean Agricultural Landscape. Conserv Biol. 2001;15: 447–456. 10.1046/j.1523-1739.2001.015002447.x

[35] Ortega CP, Ortega JC, Rapp CA, Backensto SA. Validating the Use of Artificial Nests in Predation Experiments. J Wildl Manag. 1998;62: 925–932. 10.2307/3802544

[36] Schiegg K, Eger M, Pasinelli G. Nest predation in Reed Buntings Emberiza schoeniclus: an experimental study. Ibis. 2007;149: 365–373. 10.1111/j.1474-919X.2007.00654.x

[37] Yahner RH, Voytko RA. Effects of Nest-Site Selection on Depredation of Artificial Nests. J Wildl Manag. 1989;53: 21–25. 10.2307/3801298

[38] Farnsworth GL, Simons TR. Factors Affecting Nesting Success of Wood Thrushes in Great Smoky Mountains National Park. The Auk. 1999;116: 1075–1082. 10.2307/4089686

[39] Hoset KS, Husby M. Small between-year variations in nest predation rates are not related with between-year differences in predator identity. Écoscience. 2018;25: 199–208. 10.1080/11956860.2018.1427309

[40] Schultz M, Voss J, Auer M, Carter S, Zipf A. Open land cover from OpenStreetMap and remote sensing. Int J Appl Earth Obs Geoinformation. 2017;63: 206–213. 10.1016/j.jag.2017.07.014

[41] Husby M, Hoset KS. Strong seasonal variation in nest predation rates in boreal forests. J Ornithol. 2018;159: 975–984.

[42] Andersson M, Wiklund CG. Clumping versus spacing out: Experiments on nest predation in fieldfares (*Turdus pilaris*). Anim Behav. 1978;26: 1207–1212. 10.1016/0003-3472(78)90110-0

[43] Hogstad O. Nest defence strategies in the Fieldfare *Turdus pilaris*: The responses on an avian and a mammalian predator. Ardea. 2004;92: 79–84.

[44] Sloan SS, Holmes RT, Sherry TW. Depredation rates and predators at artificial bird nests in an unfragmented northern hardwoods forest. J Wildl Manag. 1998;62: 529–539. 10.2307/3802326

[45] Collett R. Norges pattedyr [Internet]. Kristiania [Norway]: H. Aschehoug & Co. (W. Nygaard); 1911 Available: http://www.biodiversitylibrary.org/item/51536

[46] Hörnfeldt B. Long-term decline in numbers of cyclic voles in boreal Sweden: analysis and presentation of hypotheses. Oikos. 2004;107: 376–392.

[47] Bures S. High Common vole *Microtus arvalis* predation on ground-nesting bird eggs and nestlings. Ibis. 1997;139: 173–174. 10.1111/j.1474-919X.1997.tb04518.x

[48] Weidinger K. Nest predators of woodland open-nesting songbirds in central Europe. Ibis. 2009;151: 352–360. 10.1111/j.1474-919X.2009.00907.x

[49] Roos S. Functional response, seasonal decline and landscape differences in nest predation risk. Oecologia. 2002;133: 608–615. 10.1007/s00442-002-1056-8 28466159

[50] Goodrich B, Gabry J, Ali I, Brilleman S. rstanarm: Bayesian applied regression modeling via Stan. [Internet]. 2018 Available: http://mc-stan.org/

[51] R Core Team. R: A language and environment for statistical computing [Internet]. Vienna, Austria: R Foundation for Statistical Computing; 2018 Available: http://www.R-project.org

[52] Muth C, Oravecz Z, Gabry J. User-friendly Bayesian regression modeling: A tutorial with rstanarm and shinystan. Quant Methods Psychol. 2018;14: 99–119. 10.20982/tqmp.14.2.p099

[53] Gelman A, Carlin JB, Stern HS, Dunson DB, Vehtari A, Rubin DB. Bayesian Data Analysis. 3 edition Boca Raton: Chapman and Hall/CRC; 2013.

[54] Gabry J. shinystan: Interactive Visual and Numerical Diagnostics and Posterior Analysis for Bayesian Models [Internet]. 2018 Available: https://CRAN.R-project.org/package=shinystan

[55] Martin TE, Martin PR, Olson CR, Heidinger BJ, Fontaine JJ. Parental Care and Clutch Sizes in North and South American Birds. Science. 2000;287: 1482–1485. 10.1126/science.287.5457.1482 10688796

[56] Arcese P, Smith JNM, Hochachka WM, Rogers CM, Ludwig D. Stability, regulation, and the determination of abundance in an insular song sparrow population. Ecology. 1992;73: 805–822.

[57] Vander Lee BA, Lutz RS, Hansen LA, Mathews NE. Effects of supplemental prey, vegetation, and time on success of artificial nests. J Wildl Manag. 1999;63: 1299 10.2307/3802848

[58] Winter M, Johnson DH, Shaffer JA. Variability in vegetation effects on density and nesting success of grassland birds. J Wildl Manag. 2005;69: 185–197. 10.2193/0022-541X(2005)069<0185:VIVEOD>2.0.CO;2

[59] Ludwig M, Schlinkert H, Holzschuh A, Fischer C, Scherber C, Trnka A, et al Landscape-moderated bird nest predation in hedges and forest edges. Acta Oecologica-Int J Ecol. 2012;45: 50–56. 10.1016/j.actao.2012.08.008

[60] Leech SM, Leonard ML. Begging and the risk of predation in nestling birds. Behav Ecol. 1997;8: 644–646. 10.1093/beheco/8.6.644

[61] Martin TE, Scott J, Menge C. Nest predation increases with parental activity: separating nest site and parental activity effects. Proc R Soc Lond Ser B. 2000;267: 2287–2293. 10.1098/rspb.2000.1281 PMC1690815 11413645

[62] Patnode KA, White DH. Effects of habitat on avian productivity in abandoned pecan orchards in southern Georgia. J Field Ornithol. 1992;63: 77–85.

[63] Renfrew RB, Ribic CA. Grassland passerine nest predators near pasture edges identified on videotape. The Auk. 2003;120: 371–383. 10.2307/4090189

[64] Nams VO. Density-dependent predation by skunks using olfactory search images. Oecologia. 1997;110: 440–448. 10.1007/s004420050179 28307234

[65] Fontaine JJ, Martin TE. Parent birds assess nest predation risk and adjust their reproductive strategies. Ecol Lett. 2006;9: 428–434. 10.1111/j.1461-0248.2006.00892.x 16623728

[66] Paton PWC. The effect of edge on avian nest success: how strong is the evidence? Conserv Biol. 1994;8: 17–26. 10.1046/j.1523-1739.1994.08010017.x

[67] Hollander FA, Dyck HV, Martin GS, Titeux N. Nest predation deviates from nest predator abundance in an ecologically trapped bird. PLOS ONE. 2015;10: e0144098 10.1371/journal.pone.0144098 26624619 PMC4666632

[68] Burhans DE, Dearborn D, Thompson FR, Faaborg J. Factors affecting predation at songbird nests in old fields. J Wildl Manag. 2002;66: 240–249. 10.2307/3802890

[69] Cox WA, Thompson FR, Faaborg J. Species and temporal factors affect predator-specific rates of nest predation for forest songbirds in the Midwest. Auk. 2012;129: 147–155. 10.1525/auk.2012.11169

[70] Mainwaring MC, Hartley IR, Lambrechts MM, Deeming DC. The design and function of birds’ nests. Ecol Evol. 2014;4: 3909–3928. 10.1002/ece3.1054 25505520 PMC4242575

[71] Husby M. Nestling begging calls increase predation risk by corvids. Anim Biol. 2018; *advance article* 10.1163/15707563-20181058

[72] Eggers S, Griesser M, Ekman J. Predator-induced plasticity in nest visitation rates in the Siberian jay (Perisoreus infaustus). Behav Ecol. 2005;16: 309–315. 10.1093/beheco/arh163

[73] Hartley MJ, Hunter ML. A Meta-Analysis of Forest Cover, Edge Effects, and Artificial Nest Predation Rates. Conserv Biol. 1998;12: 465–469. 10.1111/j.1523-1739.1998.96373.x

[74] Jokimäki J, Huhta E. Artificial nest predation and abundance of birds along an urban gradient. The Condor. 2000;102: 838–847. 10.1650/0010-5422(2000)102[0838:ANPAAO]2.0.CO;2

[75] Martin J-L, Joron M. Nest predation in forest birds: influence of predator type and predator’s habitat quality. Oikos. 2003;102: 641–653. 10.1034/j.1600-0706.2003.12040.x

[76] Huhta E, Eramo M, Jokimäki J. Predation risk of artificial ground nests in forest stands, edges, clear-cuts, and forested corridors as an ecological indicator In: Weber RP, editor. Old-Growth Forests and Coniferous Forests: Ecology, Habitat and Conservation. New York: Nova Science Pub Inc; 2015 pp. 37–53.

[77] Huhta E, Jokimäki J, Helle P. Predation on artificial nests in a forest dominated landscape–the effects of nest type, patch size and edge structure. Ecography. 1998;21: 464–471. 10.1111/j.1600-0587.1998.tb00437.x

[78] Angelstam P. Predation on Ground-Nesting Birds’ Nests in Relation to Predator Densities and Habitat Edge. Oikos. 1986;47: 365–373. 10.2307/3565450

[79] Söderström B, Pärt T, Rydén J. Different nest predator faunas and nest predation risk on ground and shrub nests at forest ecotones: an experiment and a review. Oecologia. 1998;117: 108–118. 10.1007/s004420050638 28308476

[80] Mezquida ET, Marone L. Are results of artificial nest experiments a valid indicator of success of natural nests? Wilson Bull. 2003;115: 270–276. 10.1676/02-117

