# Supplementary material for: Are predation rates comparable between natural and artificial open-cup tree nests in boreal forest landscapes?
Source: PLoS One. 2019 Jan 9;14(1):e0210151. doi: 10.1371/journal.pone.0210151 (PMC6326507; doi:10.1371/journal.pone.0210151)
Supplement: S1 Appendix — Model building procedure with model checking and evaluation from original binomial regression model with complementary log-log link and exposure time as offset. Including r code used for analysis. (PDF) [file pone.0210151.s001.pdf]

Supplementary Appendix S1. Model building and checking

Hoset, Katrine S and Husby, Magne. Are predation rates comparable between natural and artificial open-cup tree nests in boreal forest landscapes? Submitted to PLoS ONE

12 December 2018

Data analysis

This document was produced using R Markdown in R version 3.5.1 (2018-07-02).

The nest predation data used in this study was collected both after 10 and after 25 days of nest exposure, and we want to correct for the difference in exposure time in the analysis. Some nests were checked after both 10 and 25 days exposure. Of these, nests predated before 10 days were defined to have 10 days exposure, whereas remaining nests were defined to have 25 days exposure. Such data are often fitted with a binomial regression using a complementary log-log link, combined with an offset term with the logarithm of exposure (see for example this document). The data on nest predation (available as Supplementary File S1) was sampled over three years from 5 sites, two of which were located on an island. The purpose of the study was to check whether predation rates were comparable across nest types (natural nests, natural nests with artificial eggs and artificial nests) along ecological gradients of interest, namely location along a forest edge-interior gradient and according to nest visibility. Nest visibility was originally classified as high, medium or low (see methods in main text), but the number of nests within the high visibility category was low, and only 5 and 4 of these were natural nests and natural nests with artificial eggs, respectively. In addition, we want to control for sites located on the island, for month of monitoring and taxonomic family sp.family.

```
##       year         site.no       island      forestgradient
##  Min.   :2004   Min.   : 0.000   no :102   edge      :138
##  1st Qu.:2004   1st Qu.: 1.000   yes:159   interior  : 44
##  Median :2005   Median : 1.000             transition: 79
##  Mean   :2005   Mean   : 3.418
##  3rd Qu.:2005   3rd Qu.: 9.000
##  Max.   :2006   Max.   :10.000
##
##        nesttype    month      visibility        species          sp.family
##  artificial:86   July:160   high  : 26   Artificial:86   artificial:86
##  nat.artegg:98   June: 74   low   :118   Greenfinch:66   finch     :91
##  natural   :77   May : 27   medium:117   Redwing   :60   thrush    :84
##                                          Chaffinch :18
##                                          SongThrush:14
##                                          Fieldfare : 8
##                                          (Other)   : 9
##      predT            exposure               predator    colony
##  Min.   :0.0000   Min.   :10.00   no.pred     : 40   no:261
```

```
##  1st Qu.:1.0000   1st Qu.:10.00   pred.bird   : 29
##  Median :1.0000   Median :10.00   pred.mammal :  6
##  Mean   :0.8123   Mean   :16.38   pred.unknown:186
##  3rd Qu.:1.0000   3rd Qu.:25.00
##  Max.   :1.0000   Max.   :25.00
##
##       alt.vis          predF2
##  concealed:143   Min.   :0.0000
##  visible  :118   1st Qu.:1.0000
##                  Median :1.0000
##                  Mean   :0.8123
##                  3rd Qu.:1.0000
##                  Max.   :1.0000
##
```

We wanted to fit the model as a generalised linear mixed model with site.no as random effect, and factors of interest and their interactions with nest type as fixed factors. In addition, month, island and taxonomic family was added as fixed factors, and log-exposure was added as an offset.The resulting model was too complicated to be fitted with regular frequentist methods (e.g. glmer-function from the lme4-package), so we opted to fit the model within a Bayesian framework using package rstanarm. rstanarm is a Stan-based package that allows models to be fitted using the same syntax as in the glmer-function from the lme4-package, and is developed to make Bayesian estimation routine for most common regression models (see vignettes for rstanarm). Plot functions are based on the bayesplot-package (a selection of ggplot2-based functions for plotting Bayesian models), and functions from ggplot2 can be used to enhance plots.

```
require(rstanarm)
require(ggplot2)
```

We fitted the model using function stan_glmer from the rstanarm-package. Predation events (0 or 1) was entered as the dependent variable, using a binomial distribution with a complementary log-log (cloglog) link and an offset-term with log-transformed exposure time to account for different exposure times between nests. We formally tested the probability of 0 (predT==0), i.e., the probability of nest survival. Nest type (natural, artificial and natural with artificial eggs), forest location (forest interior, edge and transition zone), visibility (concealed and visible), and the interactions between nest type and the other main effects were added as predictor variables. To control for the potential confounding effects of site location on mainland or island, nest activity month and taxonomic family (thrushes and finches), island, month, family and the interaction between family and nest type were included as predictor variables.

As weakly informative prior distributions for the intercept and the vector of regression coefficients we chose a student-t distribution with df = 3 based on information from the available documentation and vignettes for the R-package. We chose to use the default prior for the covariance structure, which is developed to be robust for common applied regression problems (ref rstanarm-paper). The covariance matrix is decomposed into a vector of variances and a correlation matrix, which summarises the covariation of slopes

and intercepts in a standardised form. Default settings describe a joint uniform prior for the correlation matrix. The vector of variances is set equal to the product of a simplex vector described by a symmetric Dirichlet prior. With the default settings of a concentration parameter at 1, this prior is jointly uniform over the space of simplex vectors. Default settings of the model produced a warning message that there were 3 divergent transitions after sampling, we therefore adjusted the step size parameter and set adapt_delta = 0.999. Plotting the posterior predicitve distribution from the model, we see that the model shows a poor fit to the distribution of the original data.

```
mod1.cloglog <-
  stan_glmer((predT==0) ~ nesttype*(forestgradient+alt.vis+sp.family) +
month+island + offset(log(exposure))+(1|site.no), data = nestpred.df, family
= binomial(link="cloglog"), prior = student_t(df = 3), prior_intercept =
student_t(df = 3), adapt_delta = 0.999)

pp_check(mod1.cloglog)
```

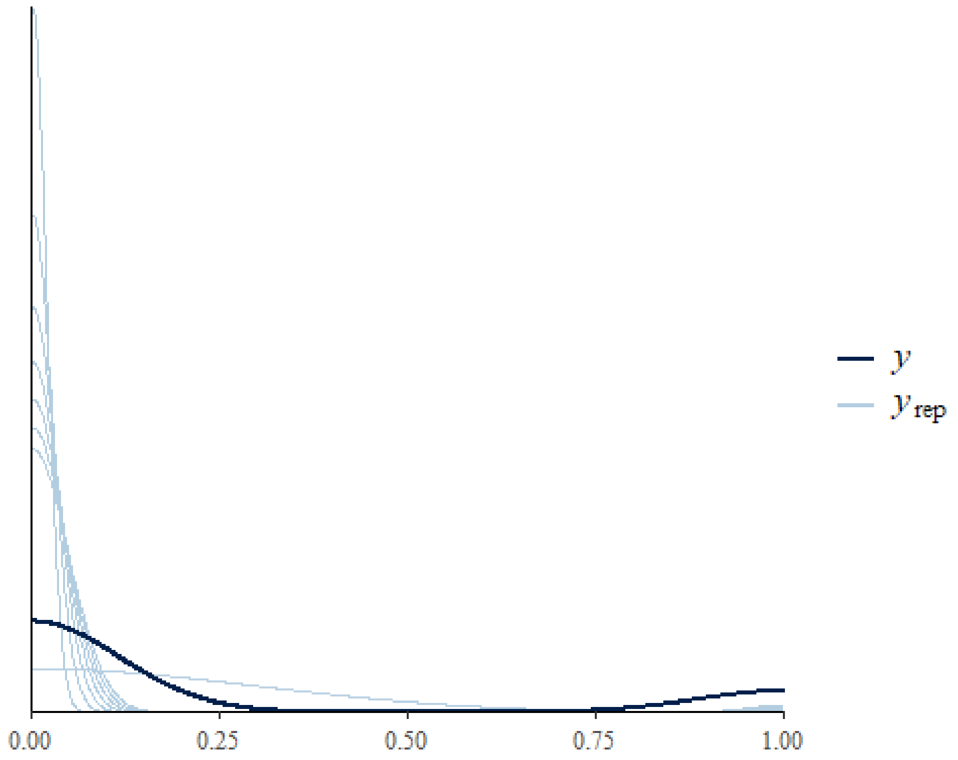

Examination of the parameters and assumptions of the model indicated that the problem was due to the inclusion of the offset-term.

```
mod1.logistic <-
  stan_glmer((predT==0) ~ nesttype*(forestgradient+alt.vis+sp.family) +
month+island + offset(log(exposure)) + (1|site.no), data = nestpred.df,
family = binomial, prior = student_t(df = 3), prior_intercept = student_t(df
= 3), adapt_delta = 0.999)
```

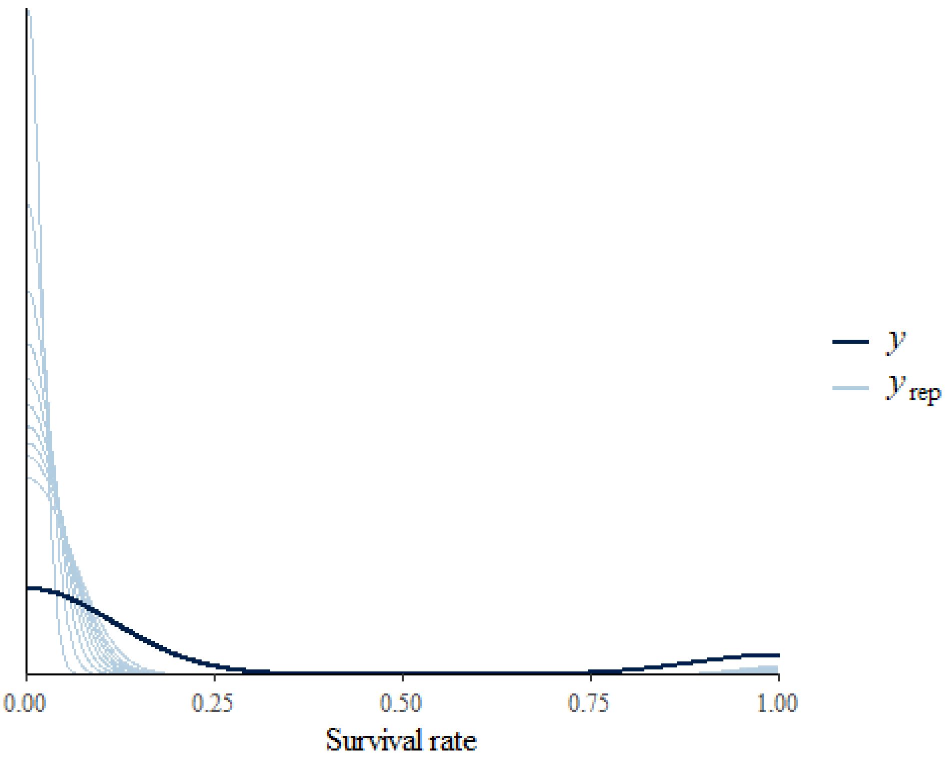

We therefore fitted the model as a logistic regression adding z-transformed exposure () as a fixed effect in the model. Posterior predictive distributions from the model were appropriate. Leave-one-out analysis (loo-function from loo-package called to by rstanarm) of the model indicated that all Pareto k estimates were ok, with 10 observations with 0.5> k < 0.7 (see for details). Numerical evaluation of the model from summary(mod2.logistic) indicated that effective sample size, Monte Carlo standard errors and ^R were acceptable, but the estimate for the intercept was unrealistically high (-4.0) and indicated a survival rate of 0.018 (or predation rate of 0.982).

```
mod2.logistic <-
  stan_glmer((predT==0) ~ nesttype*(forestgradient+alt.vis+sp.family) +
month+island+scale(exposure) + (1|site.no), data = nestpred.df, family =
binomial, prior = student_t(df = 3), prior_intercept = student_t(df = 3),
adapt_delta = 0.99)

pp_check(mod2.logistic)+xlab("Survival rate")
```

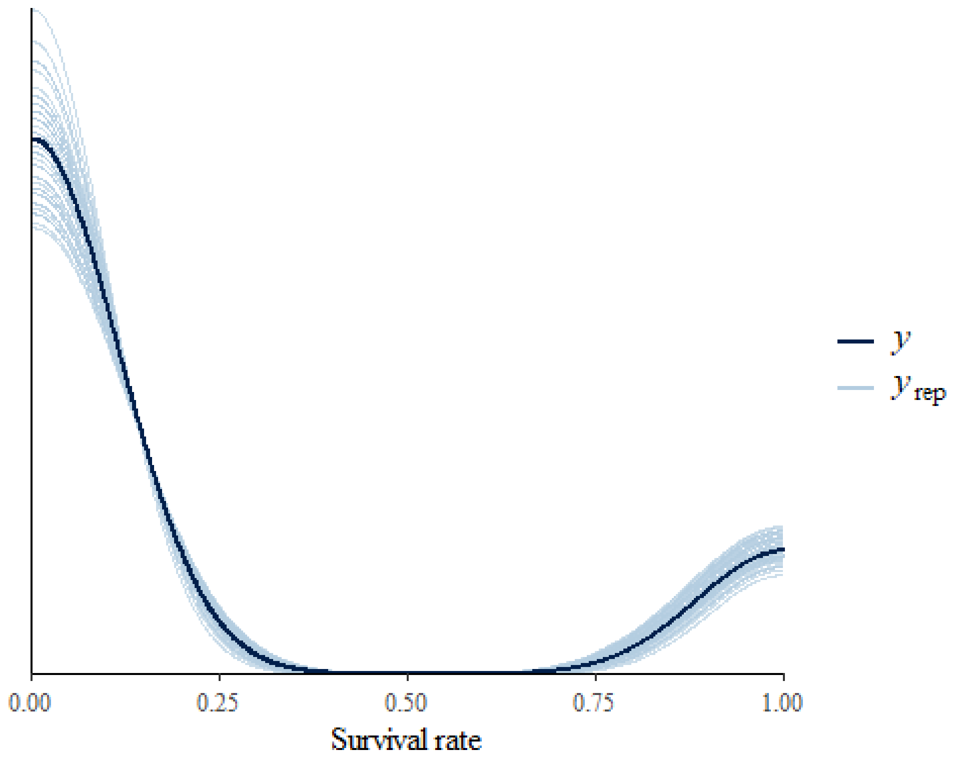


```
summary(mod2.logistic, probs = c(0.025, 0.975))

##
## Model Info:
##
##  function:     stan_glmer
##  family:       binomial [logit]
##  formula:      (predT == 0) ~ nesttype * (forestgradient + alt.vis +
sp.family) +
##     month + island + scale(exposure) + (1 | site.no)
##  algorithm:    sampling
##  priors:       see help('prior_summary')
##  sample:       4000 (posterior sample size)
##  observations: 261
##  groups:       site.no (5)
##
## Estimates:
##                                                 mean   sd    2.5%   97.5%
## (Intercept)                                    -1.4   1.1   -3.7    0.7
## nesttypenat.artegg                             -0.5   1.0   -2.6    1.5
## nesttypenatural                                -1.1   1.1   -3.4    1.0
## forestgradientinterior                         -0.3   1.3   -2.8    2.2
## forestgradienttransition                        3.4   1.1    1.4    5.8
## alt.visvisible                                 -0.8   0.9   -2.7    0.9
## sp.familyfinch                                 -0.2   0.8   -1.9    1.3
## monthJune                                      -0.8   0.9   -2.7    0.9
## monthMay                                       -1.2   1.0   -3.3    0.8
```

```
## islandyes                                    -0.5    0.9   -2.3    1.4
## scale(exposure)                               2.6    0.5    1.7    3.9
## nesttypenat.artegg:forestgradientinterior     0.1    1.3   -2.4    2.9
## nesttypenatural:forestgradientinterior        1.0    1.5   -1.8    4.0
## nesttypenat.artegg:forestgradienttransition  -4.4    1.4   -7.4   -1.8
## nesttypenatural:forestgradienttransition     -1.5    1.4   -4.3    1.0
## nesttypenat.artegg:alt.visvisible            -0.1    1.1   -2.2    2.0
## nesttypenatural:alt.visvisible               -0.7    1.1   -3.0    1.5
## nesttypenat.artegg:sp.familyfinch            -0.8    1.0   -2.6    1.1
## b[(Intercept) site.no:0]                      0.1    0.6   -1.1    1.3
## b[(Intercept) site.no:1]                     -0.1    0.6   -1.3    1.1
## b[(Intercept) site.no:2]                      0.3    0.6   -0.5    1.9
## b[(Intercept) site.no:9]                     -0.3    0.7   -2.1    0.7
## b[(Intercept) site.no:10]                     0.0    0.6   -1.2    1.2
## Sigma[site.no:(Intercept),(Intercept)]        0.6    1.3    0.0    3.5
## mean_PPD                                      0.2    0.0    0.1    0.2
## log-posterior                              -119.4    3.7 -127.5 -113.1
##
## Diagnostics:
##                                            mcse Rhat n_eff
## (Intercept)                                0.0  1.0  2066
## nesttypenat.artegg                         0.0  1.0  3039
## nesttypenatural                            0.0  1.0  2622
## forestgradientinterior                     0.0  1.0  2241
## forestgradienttransition                   0.0  1.0  1902
## alt.visvisible                             0.0  1.0  2486
## sp.familyfinch                             0.0  1.0  2863
## monthJune                                  0.0  1.0  3348
## monthMay                                   0.0  1.0  3200
## islandyes                                  0.0  1.0  2569
## scale(exposure)                            0.0  1.0  2007
## nesttypenat.artegg:forestgradientinterior   0.0  1.0  2552
## nesttypenatural:forestgradientinterior      0.0  1.0  2648
## nesttypenat.artegg:forestgradienttransition 0.0  1.0  2126
## nesttypenatural:forestgradienttransition    0.0  1.0  2470
## nesttypenat.artegg:alt.visvisible          0.0  1.0  3064
## nesttypenatural:alt.visvisible             0.0  1.0  2724
## nesttypenat.artegg:sp.familyfinch          0.0  1.0  3213
## b[(Intercept) site.no:0]                   0.0  1.0  2431
## b[(Intercept) site.no:1]                   0.0  1.0  2248
## b[(Intercept) site.no:2]                   0.0  1.0  2356
## b[(Intercept) site.no:9]                   0.0  1.0  2933
## b[(Intercept) site.no:10]                  0.0  1.0  2920
## Sigma[site.no:(Intercept),(Intercept)]     0.0  1.0  2382
## mean_PPD                                   0.0  1.0  4013
## log-posterior                              0.1  1.0  1476
##
## For each parameter, mcse is Monte Carlo standard error, n_eff is a crude
measure of effective sample size, and Rhat is the potential scale reduction
factor on split chains (at convergence Rhat=1).
```

```
gtools::inv.logit(-4, min=0, max=1)

## [1] 0.01798621

loo(mod2.logistic)

##
## Computed from 4000 by 261 log-likelihood matrix
##
##          Estimate   SE
## elpd_loo    -96.2  8.5
## p_loo        17.2  2.0
## looic       192.3 17.0
## ------
## Monte Carlo SE of elpd_loo is 0.1.
##
## Pareto k diagnostic values:
##                          Count Pct.    Min. n_eff
## (-Inf, 0.5]   (good)     260   99.6%   577
##  (0.5, 0.7]   (ok)         1    0.4%   1488
##    (0.7, 1]   (bad)        0    0.0%   <NA>
##    (1, Inf)   (very bad)   0    0.0%   <NA>
##
## All Pareto k estimates are ok (k < 0.7).
## See help('pareto-k-diagnostic') for details.
```

We then fitted the model with normal priors for the intercept and the vector of regression coefficients with mean zero and standard deviation of 1. This model provided good fit according to the posterior predictive distributions, good Pareto-k diagnostics (all k < 0.5) and good numerical evaluation (effective sample size, Monte Carlo standard error and ^R-values). Backtransformed intercept was in the range observed in the data (survival rate of 0.119, or predation rate of 0.881).

We then fitted the model with predation event as a factorial variable and formally tested the probability of predation, as this was the initial question we were interested in. And we used the shinystan-package to run full model checks on this model using the launch_shinystand function. The shintystan-package provides a graphical user interface for exploring models fit using MCMC-techniques.

```
mod3.logistic <-
  stan_glmer((predT==0) ~ nesttype*(forestgradient+alt.vis+sp.family) +
month+island+scale(exposure) + (1|site.no), data = nestpred.df, family =
binomial, prior = normal(0,1), prior_intercept = normal(0,1), adapt_delta =
0.99)

pp_check(mod3.logistic)+xlab("Survival rate")
```

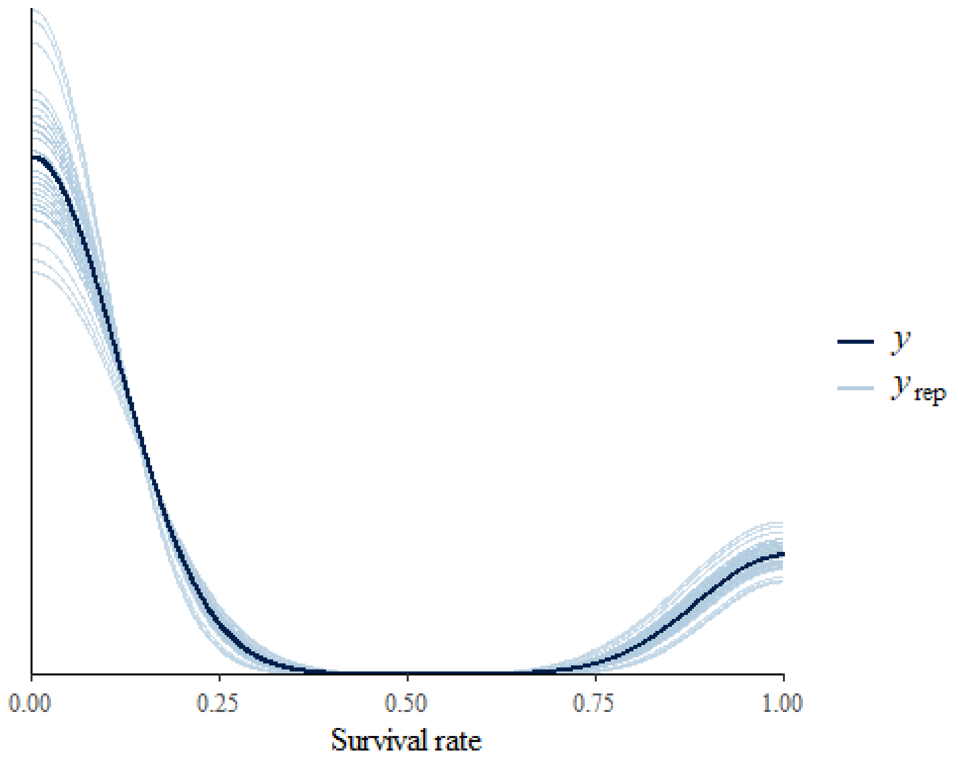


```
summary(mod3.logistic, probs = c(0.025, 0.975))

##
## Model Info:
##
##  function:     stan_glmer
##  family:       binomial [logit]
##  formula:      (predT == 0) ~ nesttype * (forestgradient + alt.vis +
sp.family) +
##     month + island + scale(exposure) + (1 | site.no)
##  algorithm:    sampling
##  priors:       see help('prior_summary')
##  sample:       4000 (posterior sample size)
##  observations: 261
##  groups:       site.no (5)
##
## Estimates:
##                                                 mean   sd    2.5%   97.5%
## (Intercept)                                     -0.8   0.7  -2.1    0.6
## nesttypenat.artegg                              -0.2   0.6  -1.4    0.9
## nesttypenatural                                 -0.7   0.6  -2.0    0.5
## forestgradientinterior                          -0.1   0.7  -1.4    1.2
## forestgradienttransition                         1.4   0.5   0.3    2.3
## alt.visvisible                                  -0.6   0.5  -1.7    0.4
## sp.familyfinch                                  -0.4   0.5  -1.4    0.6
## monthJune                                       -0.3   0.6  -1.4    0.8
## monthMay                                        -0.6   0.6  -1.8    0.7
```

```
## islandyes                                      -0.2    0.7   -1.5    1.1
## scale(exposure)                                 1.4    0.2    0.9    1.8
## nesttypenat.artegg:forestgradientinterior       0.4    0.7   -1.0    1.8
## nesttypenatural:forestgradientinterior          0.4    0.8   -1.2    1.9
## nesttypenat.artegg:forestgradienttransition    -1.5    0.7   -2.9   -0.2
## nesttypenatural:forestgradienttransition       -0.2    0.7   -1.6    1.3
## nesttypenat.artegg:alt.visvisible              -0.3    0.7   -1.6    1.0
## nesttypenatural:alt.visvisible                 -0.4    0.7   -1.8    0.9
## nesttypenat.artegg:sp.familyfinch              -0.3    0.6   -1.6    1.0
## b[(Intercept) site.no:0]                       -0.1    0.5   -1.4    0.8
## b[(Intercept) site.no:1]                       -0.4    0.6   -1.8    0.4
## b[(Intercept) site.no:2]                        0.2    0.5   -0.7    1.3
## b[(Intercept) site.no:9]                       -0.4    0.7   -2.2    0.5
## b[(Intercept) site.no:10]                      -0.1    0.5   -1.4    0.8
## Sigma[site.no:(Intercept),(Intercept)]          0.6    1.1    0.0    3.4
## mean_PPD                                        0.2    0.0    0.1    0.3
## log-posterior                                -129.9    3.8 -138.3 -123.3
##
## Diagnostics:
##                                              mcse Rhat n_eff
## (Intercept)                                  0.0  1.0  3041
## nesttypenat.artegg                           0.0  1.0  4050
## nesttypenatural                              0.0  1.0  3687
## forestgradientinterior                       0.0  1.0  3471
## forestgradienttransition                     0.0  1.0  4215
## alt.visvisible                               0.0  1.0  3684
## sp.familyfinch                               0.0  1.0  3756
## monthJune                                    0.0  1.0  3946
## monthMay                                     0.0  1.0  3876
## islandyes                                    0.0  1.0  3028
## scale(exposure)                              0.0  1.0  4511
## nesttypenat.artegg:forestgradientinterior    0.0  1.0  3884
## nesttypenatural:forestgradientinterior       0.0  1.0  4139
## nesttypenat.artegg:forestgradienttransition 0.0  1.0  3973
## nesttypenatural:forestgradienttransition     0.0  1.0  4850
## nesttypenat.artegg:alt.visvisible            0.0  1.0  4125
## nesttypenatural:alt.visvisible               0.0  1.0  4746
## nesttypenat.artegg:sp.familyfinch            0.0  1.0  4060
## b[(Intercept) site.no:0]                     0.0  1.0  2353
## b[(Intercept) site.no:1]                     0.0  1.0  1368
## b[(Intercept) site.no:2]                     0.0  1.0  2972
## b[(Intercept) site.no:9]                     0.0  1.0  1777
## b[(Intercept) site.no:10]                    0.0  1.0  2662
## Sigma[site.no:(Intercept),(Intercept)]       0.0  1.0  1926
## mean_PPD                                     0.0  1.0  4253
## log-posterior                                0.1  1.0  1517
##
## For each parameter, mcse is Monte Carlo standard error, n_eff is a crude
measure of effective sample size, and Rhat is the potential scale reduction
factor on split chains (at convergence Rhat=1).
```

```
gtools::inv.logit(-2, min=0, max=1)

## [1] 0.1192029

loo(mod3.logistic)

##
## Computed from 4000 by 261 log-likelihood matrix
##
##            Estimate   SE
## elpd_loo     -97.6  7.6
## p_loo         11.6  1.1
## looic        195.2 15.3
## ------
## Monte Carlo SE of elpd_loo is 0.1.
##
## All Pareto k estimates are good (k < 0.5).
## See help('pareto-k-diagnostic') for details.

mod4.logistic <-
  stan_glmer(predF2 ~ nesttype*(forestgradient+alt.vis+sp.family) +
month+island+scale(exposure) + (1|site.no), data = nestpred.df, family =
binomial, prior = normal(0,1), prior_intercept = normal(0,1), adapt_delta =
0.99)

pp_check(mod4.logistic)+xlab("Predation rate")
```

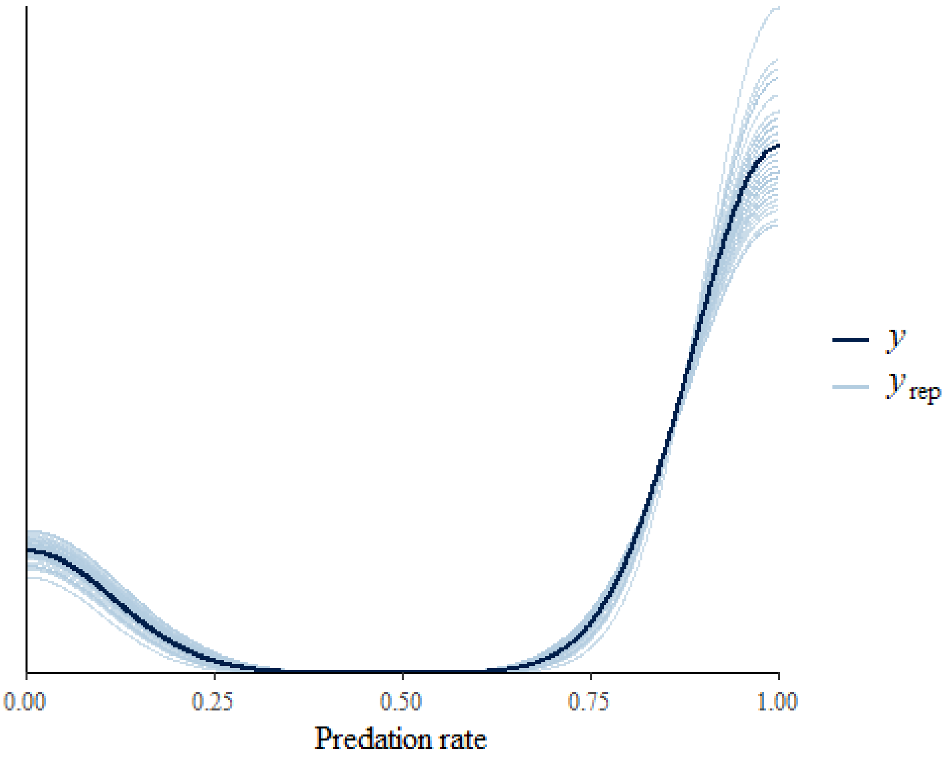


```
summary(mod4.logistic, digits=3, probs = c(0.025, 0.975))
```

```
##
## Model Info:
##
##  function:     stan_glmer
##  family:       binomial [logit]
##  formula:      predF2 ~ nesttype * (forestgradient + alt.vis + sp.family)
+
##     month + island + scale(exposure) + (1 | site.no)
##  algorithm:    sampling
##  priors:       see help('prior_summary')
##  sample:       4000 (posterior sample size)
##  observations: 261
##  groups:       site.no (5)
##
## Estimates:
##                                                mean     sd       2.5%
## (Intercept)                                     0.817    0.676   -0.551
## nesttypenat.artegg                              0.236    0.600   -0.895
## nesttypenatural                                 0.708    0.628   -0.580
## forestgradientinterior                          0.136    0.643   -1.141
## forestgradienttransition                       -1.341    0.507   -2.359
## alt.visvisible                                  0.638    0.537   -0.384
## sp.familyfinch                                  0.353    0.538   -0.694
## monthJune                                       0.310    0.559   -0.764
## monthMay                                        0.538    0.626   -0.665
## islandyes                                       0.240    0.647   -1.133
## scale(exposure)                                -1.389    0.235   -1.871
## nesttypenat.artegg:forestgradientinterior      -0.434    0.712   -1.842
## nesttypenatural:forestgradientinterior         -0.376    0.785   -1.928
## nesttypenat.artegg:forestgradienttransition     1.536    0.681    0.187
## nesttypenatural:forestgradienttransition        0.148    0.706   -1.257
## nesttypenat.artegg:alt.visvisible               0.304    0.658   -0.976
## nesttypenatural:alt.visvisible                  0.444    0.680   -0.858
## nesttypenat.artegg:sp.familyfinch               0.339    0.628   -0.892
## b[(Intercept) site.no:0]                        0.109    0.514   -0.778
## b[(Intercept) site.no:1]                        0.375    0.550   -0.381
## b[(Intercept) site.no:2]                       -0.230    0.479   -1.317
## b[(Intercept) site.no:9]                        0.418    0.660   -0.428
## b[(Intercept) site.no:10]                       0.103    0.522   -0.864
## Sigma[site.no:(Intercept),(Intercept)]          0.556    1.010    0.001
## mean_PPD                                        0.805    0.030    0.747
## log-posterior                                -129.673    3.838 -138.192
##                                                97.5%
## (Intercept)                                     2.099
## nesttypenat.artegg                              1.407
## nesttypenatural                                 1.942
## forestgradientinterior                          1.371
## forestgradienttransition                       -0.361
## alt.visvisible                                  1.718
## sp.familyfinch                                  1.396
```

```
## monthJune                                       1.406
## monthMay                                        1.806
## islandyes                                       1.457
## scale(exposure)                                -0.956
## nesttypenat.artegg:forestgradientinterior       0.974
## nesttypenatural:forestgradientinterior          1.214
## nesttypenat.artegg:forestgradienttransition     2.893
## nesttypenatural:forestgradienttransition        1.560
## nesttypenat.artegg:alt.visvisible               1.576
## nesttypenatural:alt.visvisible                  1.806
## nesttypenat.artegg:sp.familyfinch               1.567
## b[(Intercept) site.no:0]                        1.398
## b[(Intercept) site.no:1]                        1.778
## b[(Intercept) site.no:2]                        0.620
## b[(Intercept) site.no:9]                        2.164
## b[(Intercept) site.no:10]                       1.322
## Sigma[site.no:(Intercept),(Intercept)]          3.190
## mean_PPD                                        0.862
## log-posterior                                -123.188
##
## Diagnostics:
##                                             mcse  Rhat  n_eff
## (Intercept)                                 0.012 1.000 3203
## nesttypenat.artegg                          0.010 0.999 3447
## nesttypenatural                             0.011 1.001 3110
## forestgradientinterior                      0.011 1.001 3701
## forestgradienttransition                    0.008 1.000 3560
## alt.visvisible                              0.009 1.000 3657
## sp.familyfinch                              0.009 1.000 3404
## monthJune                                   0.009 1.001 3478
## monthMay                                    0.010 1.001 3736
## islandyes                                   0.013 1.003 2567
## scale(exposure)                             0.004 1.000 3482
## nesttypenat.artegg:forestgradientinterior   0.011 1.000 4346
## nesttypenatural:forestgradientinterior      0.012 1.000 3992
## nesttypenat.artegg:forestgradienttransition 0.011 1.001 4014
## nesttypenatural:forestgradienttransition    0.011 1.000 4456
## nesttypenat.artegg:alt.visvisible           0.011 0.999 3427
## nesttypenatural:alt.visvisible              0.011 1.000 3915
## nesttypenat.artegg:sp.familyfinch           0.010 1.000 3634
## b[(Intercept) site.no:0]                    0.012 1.002 1948
## b[(Intercept) site.no:1]                    0.015 1.003 1421
## b[(Intercept) site.no:2]                    0.010 1.000 2524
## b[(Intercept) site.no:9]                    0.015 1.001 1841
## b[(Intercept) site.no:10]                   0.010 0.999 2908
## Sigma[site.no:(Intercept),(Intercept)]      0.024 1.004 1750
## mean_PPD                                    0.000 1.000 4006
## log-posterior                               0.106 1.004 1317
##
## For each parameter, mcse is Monte Carlo standard error, n_eff is a crude
```

```
measure of effective sample size, and Rhat is the potential scale reduction
factor on split chains (at convergence Rhat=1).

gtools::inv.logit(2.0, min = 0, max = 1)

## [1] 0.8807971

launch_shinystan(mod4.logistic)
```

As a final step, we plotted the estimated parameter effects using MCMCplot-function from the MCMCvis-package and calculated posterior probabilities for effects being smaller or larger than zero for all fixed effects.

```
fit.mod4<-as.matrix(mod4.logistic)
param.mod4<-
c("(Intercept)","nesttypenat.artegg","nesttypeart.syst","forestgradienttransi
tion","forestgradientedge","alt.visvisible","sp.familythrush","monthJune","mo
nthJuly","islandyes","scale(exposure)","nesttypenat.artegg:forestgradienttran
sition","nesttypeart.syst:forestgradienttransition","nesttypenat.artegg:fores
tgradientedge","nesttypeart.syst:forestgradientedge","nesttypenat.artegg:alt.
visvisible","nesttypeart.syst:alt.visvisible","nesttypenat.artegg:sp.familyth
rush")
lab.mod4<-
c("Intercept","nesttype_nat.artegg","nesttype_art","forestgradient_transition
","forestgradient_edge","visibility_visible","family_thrush","month_June","mo
nth_July","island_yes","z-
transf_exposure","nesttype_nat.artegg:forestgradient_transition","nesttype_ar
t:forestgradient_transition","nesttype_nat.artegg:forestgradient_edge","nestt
ype_art:forestgradient_edge","nesttype_nat.artegg:visibility_visible","nestty
pe_art:visibility_visible","nesttype_nat.artegg:family_thrush")
MCMCvis::MCMCplot(fit.mod4,
                  params = param.mod4,
                  xlim = c(-5, 5),
                  xlab = 'Estimates for logit(predation rate)',
                  labels = lab.mod4,
                  guide_axis = TRUE)

postNatartegg<-as.matrix(rev3.stan3p, pars="nesttypenat.artegg")
postArt<-as.matrix(rev3.stan3p, pars="nesttypeart.syst")
postTrans<-as.matrix(rev3.stan3p, pars="forestgradienttransition")
postEdge<-as.matrix(rev3.stan3p, pars="forestgradientedge")
postVis<-as.matrix(rev3.stan3p, pars="alt.visvisible")
postThrush<-as.matrix(rev3.stan3p, pars="sp.familythrush")
postJune<-as.matrix(rev3.stan3p, pars="monthJune")
postJuly<-as.matrix(rev3.stan3p, pars="monthJuly")
postIsland<-as.matrix(rev3.stan3p, pars="islandyes")
postNatart.Trans<-as.matrix(rev3.stan3p,
pars="nesttypenat.artegg:forestgradienttransition")
postNatart.Edge<-as.matrix(rev3.stan3p,
pars="nesttypenat.artegg:forestgradientedge")
postArt.Trans<-as.matrix(rev3.stan3p,
```

```
pars="nesttypeart.syst:forestgradienttransition")
postArt.Edge<-as.matrix(rev3.stan3p,
pars="nesttypeart.syst:forestgradientedge")
postNatart.Vis<-as.matrix(rev3.stan3p,
pars="nesttypenat.artegg:alt.visvisible")
postArt.vis<-as.matrix(rev3.stan3p, pars="nesttypeart.syst:alt.visvisible")
postNatart.thrush<-as.matrix(rev3.stan3p,
pars="nesttypenat.artegg:sp.familythrush")
postExposure<-as.matrix(rev3.stan3p, pars="scale(exposure)")
postIntercept<-as.matrix(rev3.stan3p, pars="(Intercept)")

#Posterior predictions/probability effect is smaller/larger than zero:
mean(postNatartegg<0);mean(postNatartegg>0)
mean(postArt<0);mean(postArt>0)
mean(postTrans<0);mean(postTrans>0)
mean(postEdge<0);mean(postEdge>0)
mean(postVis<0);mean(postVis>0)
mean(postThrush<0);mean(postThrush>0)
mean(postJune<0);mean(postJune>0)
mean(postJuly<0);mean(postJuly>0)
mean(postIsland<0);mean(postIsland>0)
mean(postNatart.Trans<0);mean(postNatart.Trans>0)
mean(postNatart.Edge<0);mean(postNatart.Edge>0)
mean(postArt.Trans<0);mean(postArt.Trans>0)
mean(postArt.Edge<0);mean(postArt.Edge>0)
mean(postNatart.Vis<0);mean(postNatart.Vis>0)
mean(postArt.vis<0);mean(postArt.vis>0)
mean(postNatart.thrush<0);mean(postNatart.thrush>0)
mean(postExposure<0);mean(postExposure>0)
mean(postIntercept<0);mean(postIntercept>0)
```
