# Supplementary material for: Are predation rates comparable between natural and artificial open-cup tree nests in boreal forest landscapes?
Source: PLoS One. 2019 Jan 9;14(1):e0210151. doi: 10.1371/journal.pone.0210151 (PMC6326507; doi:10.1371/journal.pone.0210151)
Supplement: S1 Fig — Effect sizes of predictor variables explaining variation in nest predation rates based on dataset where all nests observed at both 10 and 25 days were categorized as having 25 days exposure time. (DOCX) [file pone.0210151.s003.docx]

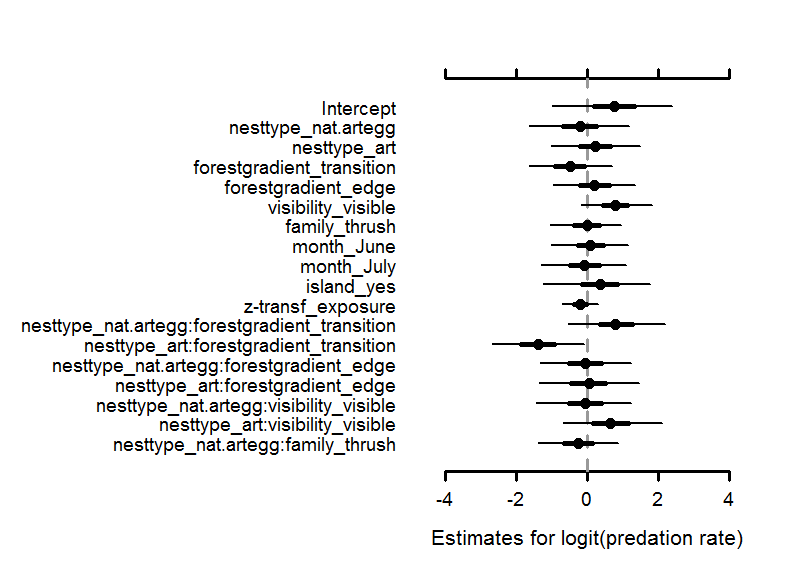


**S1 Figure. Dependency of exposure time effect on categorization of exposure time in nests observed at both 10 and 25 days.** Effect sizes of predictor variables explaining variation in nest predation rates based on dataset where all nests observed at both 10 and 25 days were categorized as having 25 days exposure time.
