# Supplementary material for: Are predation rates comparable between natural and artificial open-cup tree nests in boreal forest landscapes?
Source: PLoS One. 2019 Jan 9;14(1):e0210151. doi: 10.1371/journal.pone.0210151 (PMC6326507; doi:10.1371/journal.pone.0210151)
Supplement: S1 Table — The table highlights how nest belonging to different nest type categories are distributed between 10 and 25 days exposure time in the dataset used for analysis in the paper, and for two alternative datasets where all nests observed after both 10 and 25 days are defined as having exposure time of either 10 days (Adjusted exposure time 10 days) or 25 days (Adjusted exposure time 25 days). See S1 Fig how the difference in categorization affects predictor estimates, and especially the effect of z-transformed exposure time. (DOCX) [file pone.0210151.s004.docx]

**S1 Table. Distribution of nest types according to exposure time.** The table highlights how nest belonging to different nest type categories are distributed between 10 and 25 days exposure time in the dataset used for analysis in the paper, and for two alternative datasets where all nests observed after both 10 and 25 days are defined as having exposure time of either 10 days (Adjusted exposure time 10 days) or 25 days (Adjusted exposure time 25 days). See S1 Figure how the difference in categorization affects predictor estimates, and especially the effect of z-transformed exposure time.

|  | **Original dataset** | | **Adjusted exposure time 10 days** | | **Adjusted exposure time 25 days** | |
| --- | --- | --- | --- | --- | --- | --- |
| **Nest type** | **10 days** | **25 days** | **10 days** | **25 days** | **10 days** | **25 days** |
| Natural | 31 | 46 | 34 | 43 | 1 | 76 |
| Natural with artificial egg | 42 | 56 | 98 | 0 | 0 | 98 |
| Artificial | 77 | 9 | 86 | 0 | 45 | 41 |
